# Cementing Partnerships: Applying a Network-Mapping Tool in Tajikistan

**DOI:** 10.3389/fpubh.2021.585806

**Published:** 2021-02-18

**Authors:** Nazira R. Muhamedjonova, Jonathan Watkins, Sayora I. Nazarova, P. A. Holding

**Affiliations:** ^1^HealthProm, Dushanbe, Tajikistan; ^2^HealthProm, London, United Kingdom; ^3^Saving Brains Collaborative Learning Team, Mombasa, Kenya

**Keywords:** network-mapping, care partnerships, child support, communication, inclusion

## Abstract

**Background:** This article describes the integration of an innovative network-mapping tool within a monitoring, evaluation and learning system. We describe how it serves to strengthen vulnerable families to care for their children. We discuss the use of this tool as part of the process of measurement for change in the preparation for the sustainable scaling of programme implementation. Tajikistan has a legacy of Soviet-style institutional care of children. Traditionally, very young children separated from their families have been cared for in institutional baby homes. This research is part of a wider project to transform these baby homes into community-oriented Family and Child Support Centres.

**Methods:** We mapped the networks of child support experienced by parents and service providers. We used interactive, semi-structured interviews, and the tool evolved through an iterative process. We generated data to describe the connexions between children, families, professionals and supporting organisations. The resulting information revealed strengths and weaknesses in support provided, attitudes and perceptions towards the quality of the support as well as identifying processes through which changes strengthening the system can be stimulated.

**Results:** The data showed that the main support for children comes from within their immediate household, but, over time, more distal support gained value. Variation in the networks of support related to gender, specific subgroups of need and location. Gender was the most influential determinant of patterns of support. Mothers' knowledge of service provision, represented by a greater number and variety of contacts on their network-maps, was more diverse than fathers'. In contrast, fathers' more limited networks showed connexions to individuals and organisations with potentially more powerful decision-making roles. Participation in the discussions around the network-mapping contributed towards a change in the use of data and evidence in the implementation team.

**Conclusions:** Network-mapping is a valuable and adaptable tool that feeds into monitoring and evaluation at multiple levels. The process reveals the nature and extent of relationships of support for childcare and protection. It exposes the changes in these networks over time. Both the information provided and the process of collection can enrich care plans, create links within the network and inform decision-making that improves efficacy of delivery as we move to scale.

## Introduction

Keeping families together creates continuity in the support necessary for a child to make a successful transition to healthy adulthood (1). The UK NGO HealthProm^1^ began work in Tajikistan in 2006 with a mandate from the Dushanbe City Health Department. The overall objective was to transform the institutional focus of infant support from the Baby Homes into community-based family support networks. To stimulate radical transformation of a system requires careful, considered planning at multiple levels (2, 3).

HealthProm has worked on the Putting Families First (PFF) project in collaboration with government departments, local NGO partners, staff of the Baby Homes and international partners to establish family support services next to all the Baby Homes. The specific objectives are to provide vulnerable families with a more effective alternative to institutional care for their young children; to strengthen practises of nurturing care and child protection; and, to build alternative family based care for children who cannot live with their birth parents.

Through this partnership centres have been established that offer a range of services to support families at risk of abandoning a child. Individualised programmes meet the needs of families either facing difficult life situations or caring for children living with a disability. Services offered include physical and speech therapy, parent training, social welfare and psychological support. We have gained increasing support from the community. In 2019 we received official recognition of the shift towards community oriented child support. The Ministry of Health and Social Protection of the Population, and Local Government Authorities agreed new regulations to merge the Family Support Centres and the Baby Homes forming new community-oriented Family and Child Support Centres.

This article describes how we used a network-mapping tool to reveal the relationships that impact upon the community based system for long-term care of vulnerable children. The tool forms an integral part of monitoring and evaluating the theory of change that guides the PFF programme. We sought proof of the concept that replacing institutional care in Tajikistan with an innovative set of interventions has a positive effect on child development. Central to the theory of change is strengthening families to support their children's development. A tool that examines social interactions and social support was seen to be an essential part of the suite of tools required to produce evidence of this pathway (4). The information provided guides project development and steers its sustainable implementation at scale.

## Context

At independence in 1991 the country inherited a state-run care system for children that was almost entirely institution based. Alternative support systems provided by the non-state sector were very limited and uncoordinated. Very young children whose families struggled to care for their children because of social, economic pressures, or who felt unable to manage their child's disability were placed in state-run Baby Homes. In law, these Baby Homes were closed institutions. This restricted family contact, and left the care of children to the staff who worked on a rota system and had inconsistent contact with their charges. Although children in the Baby Homes were provided with basic needs, they were exposed to the potential for significantly disrupted attachment (5).

**Figure 1:**
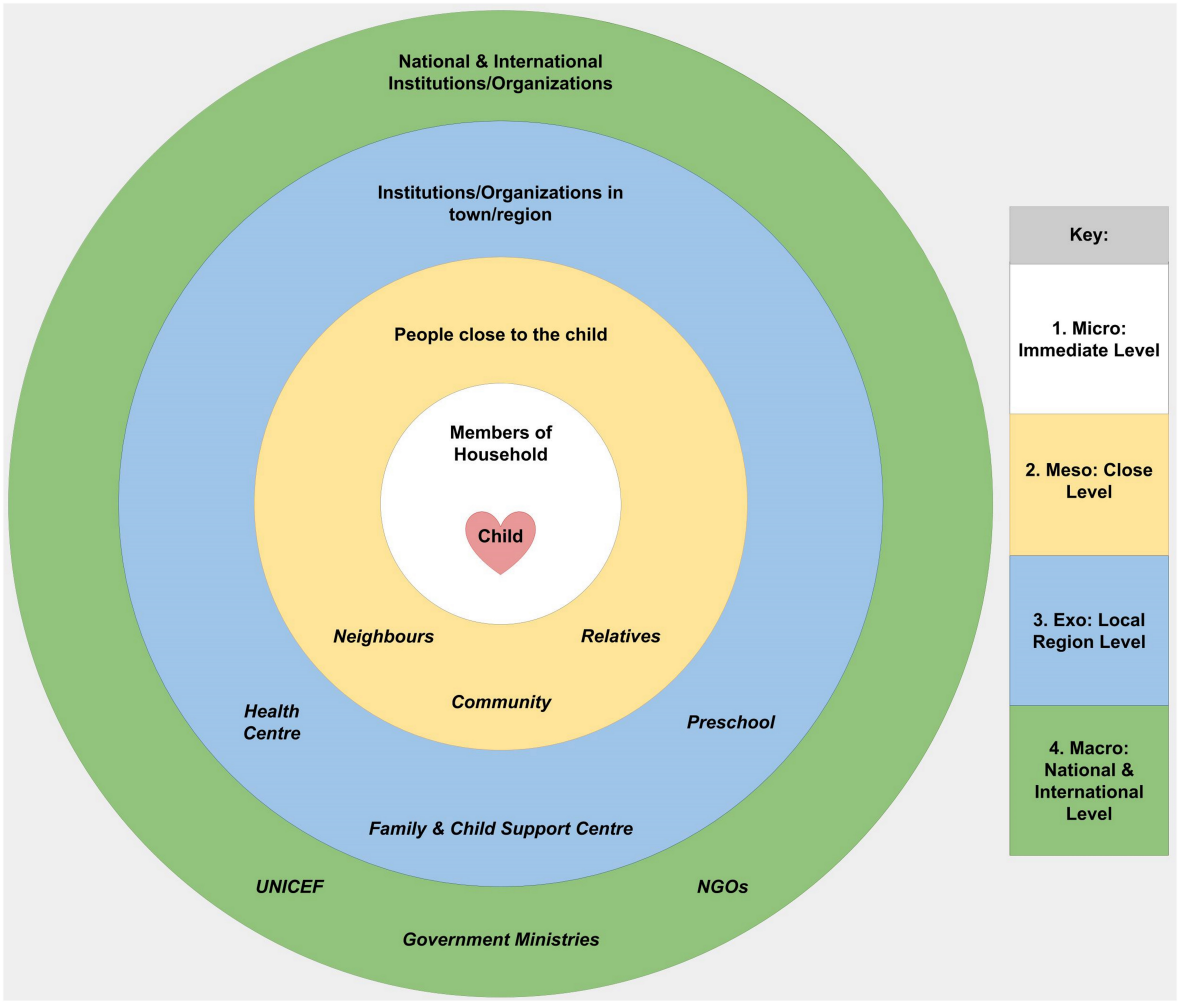
Bronfenbrenner's analytic model and its application in the context of Tajikistan.

Tajikistan is predominantly a traditional rural society (6) where children live as part of an extended family household. The micro-level of a baby and parent dyad and the meso level of multigenerational kin tend to be merged into wider family units (7) (See Figure 1). Several related families often live around a communal courtyard. An informal network extends beyond kin into the local farming community where households merge and interact through shared economic and cultural activities.

This traditional model does not hold for the 27.3% of the population who live in urban areas^2^ and for families who have been excluded socially. Mothers and children are at risk of exclusion when a child's father remarries or moves abroad to work. In 2019, 484,000 Tajiks migrated abroad for work.^3^ With these more vulnerable children and parents, there is a greater barrier to wider family support as the parent/child dyad lives isolated from extended family, usually in an apartment or less stable urban locations.

The local environment comprises a range of local government administration, health and education services. At the most grass-roots level of local government, the Mahala Committees address street-level matters. Access to government services in health and education is dependent upon appropriate registration. Unregistered families are excluded. Local Government Authorities provide a basic safety net of social protection through Child Rights Units. Families can receive a degree of social protection from the state, such as small pensions, but not a comprehensive and coordinated network of care and support. Practical advice for families, parenting programmes and child development services are only provided by a few non-governmental organisations, such as HealthProm's local partners.

We took Bronfenbrenner's Ecological Systems Theory (EST) (8) as our conceptual framework and used network-mapping to draw out information on the support provided at different degrees of separation from the family. We applied this model through multi-layered maps to explore the nature and degree of parent and child interactions within the social system unique to Tajikistan.

## Key Programmatic Elements

**Figure 2:**
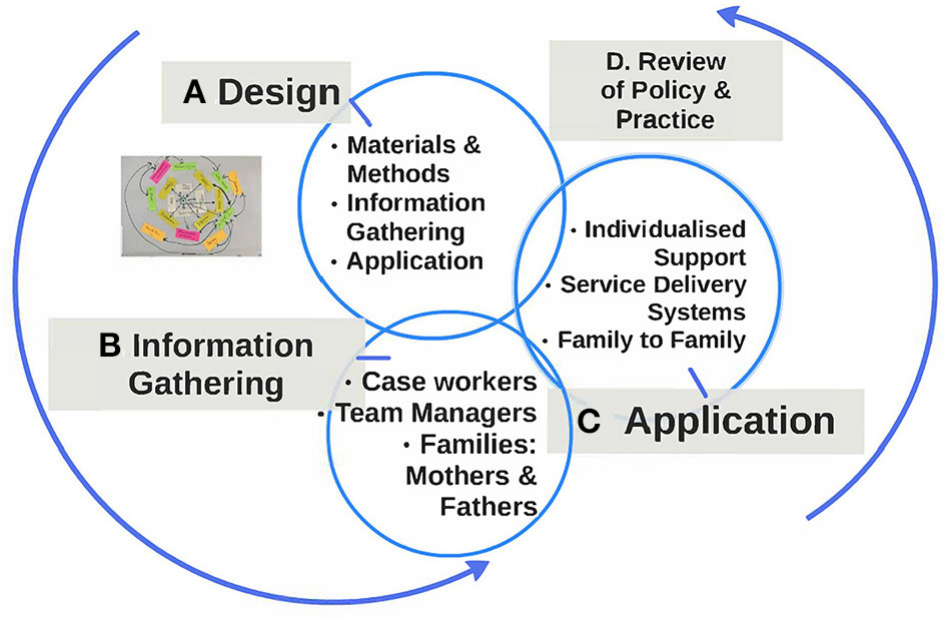
Network-maps -the details of the programmatic elements.

To describe in detail the networks of support and to stimulate conversations around providing nurturing care we followed a process outlined in Figure 2. This captures the cyclical, interconnected, nature of the activities labelled anti clockwise as Stage A (Design) to Stage D (Review). While each activity focus depends upon completing a previous stage, we also drew upon the experience of later stages to refine and improve our procedures (9).

Thus, the formative component of the project Stage B (Information Gathering) both informs the next step Stage C (Application) and is informed by it. Likewise Stage D (the Review of Policy and Practise), both influences decision making at the meso and macro level, as well as guiding future Design (Stage A) as the project transitions to scale.

### Stage A. The Process of Design

The network-mapping sessions were designed to strengthen the networks of support in multiple ways. Strengths, weaknesses and suggested improvements in the existing networks were to be identified using the maps. The sessions were to provide a forum for sharing knowledge and making bonds between families in need. The changes observed over time tracked the influence of the PFF intervention on the support network. By sharing information on patterns and trends with policy-makers, we intended to strengthen communication channels between the different levels of support. Ethical approval for this process was granted by the Ministry of Health, and informed consent obtained from participants prior to each session.

The initial objective was to develop a process to monitor systematically the childcare networks of families receiving support from the PFF programme. Two monitoring and evaluation consultants, one local and one international, guided the design phase. Working with the management and service delivery teams, materials and methods were evaluated to ensure the language used was accessible and that processes were feasible to administer and engaging for participants. The process of design was guided by the following questions, “Are participants actively contributing to the sessions?” And, “Are we able to capture the contributions systematically and accurately?”

We selected *Network-maps*^4^ (10) as the template for our information gathering process, making modifications to fit our focus and our context. Given that adaptations were made, we have chosen to use the term network-mapping to describe the process utilised in this study. Modifications included building into the data capture process five levels of support suggested by the Bronfenbrenner EST model (see Figure 1 above). Participants were asked to reflect upon support/services they received at each of the five levels described, the Immediate, Close, Regional, National, and International. We found that participants related with ease to this framework as a way of organising their experiences.

**Figure 3:**
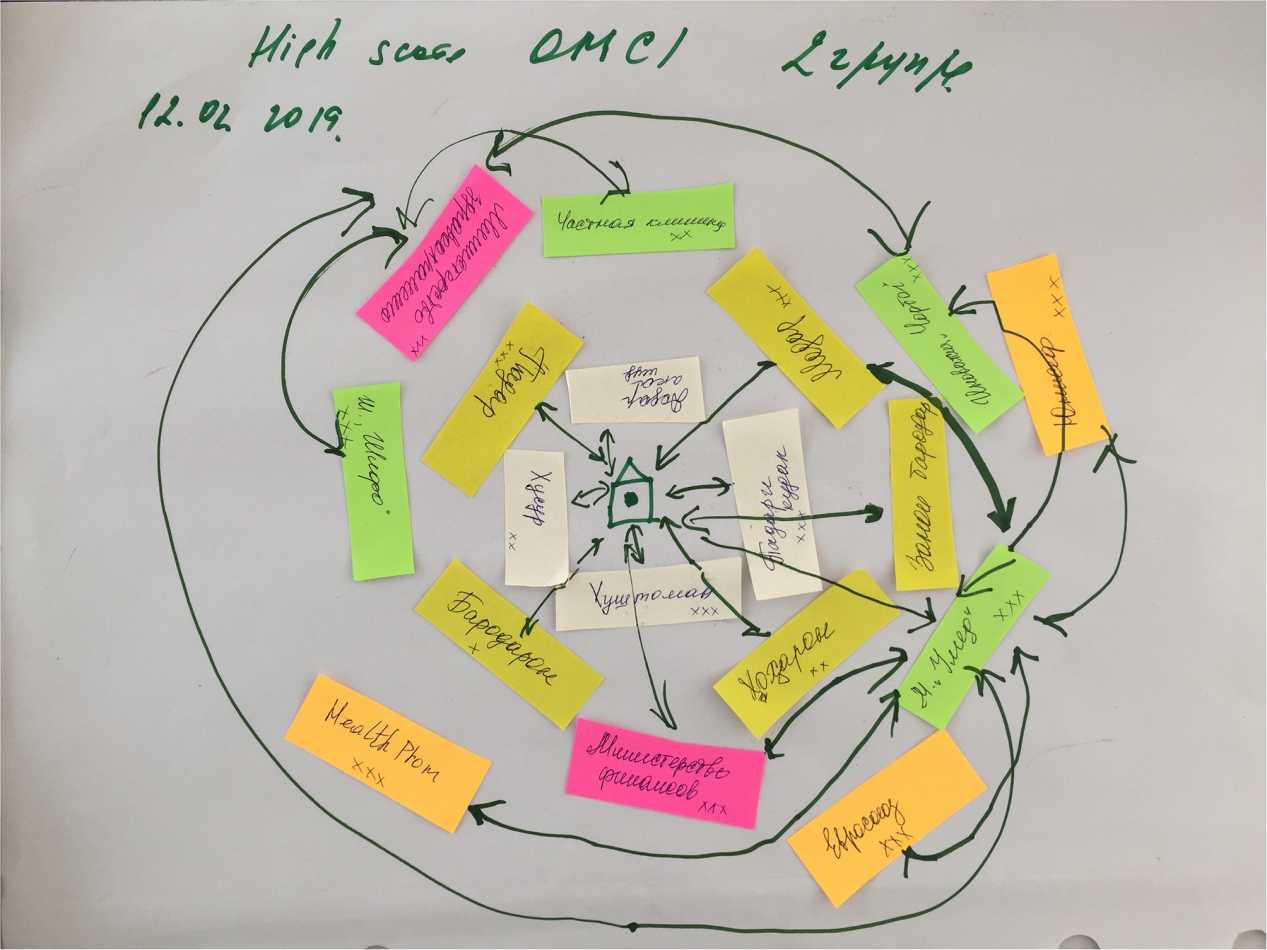
An example net-map.

The maps summarised the collective experience of the participants, derived through consensus within each group. Information shared by participants was first captured on coloured sticky notes. The participants arranged these notes on a single sheet. Information on attributed value and motivation were added to the sticky notes. The sticky notes meant that they could, in the course of the conversation, return to the details, make adjustments, ask questions and add their own comments and contributions. The maps produced provided participants with a concrete way to see how their network was built. An example is provided in Figure 3 below.

The network-mapping sessions also provided the opportunity for participants to share their personal experiences as a caregiver, as well as their experience of the PFF intervention and of other services. We captured these narratives in the meeting notes.

We designed separate data capture forms to provide a systematic framework for making comparisons between groups and sub groups of participants.^5^ These forms captured details on the following characteristics of support:

1. **Source of support**: defined by their role or organisation name.
2. **Type of support**: categorised as Services Directly to the Child, Integrating the Child into Community and or Family Systems, Skill Development, Resources Supplied, the Promotion of Child Protection.
3. **Perceived value/level of importance**: categorised as minimal, some value, very important/valued.
4. **Motivation attributed to the provider**: categorised as Social Responsibility (*because they care about the child*), Social Change (*because they want to create change*), Personal or Professional Responsibility (*in the course of meeting their expected role or responsibilities*).

These characteristics were rated according to categories suggested by responses given in the first round of sessions, and validated through application in later rounds. Data tables provided a summary of the ratings across each characteristic.

As this was an experimental process we chose to use one key facilitator to run all of the sessions. This had the advantage that diverse groups of informants were managed in a consistent way. It also provided a coordinated approach to data capture, analysis, application and reflection. Using a facilitator not directly involved in service delivery or programme management was seen as a key factor in stimulating the high degree of candidness with which parents shared their opinions and feelings. However, the key facilitator had to cover large distances over remote mountainous terrain,^6^ and this placed limitations on the timing of the rounds of network-mapping sessions. The facilitator found the tool increasingly easy to apply.

The perspective of the primary or key caregiver was the main focus of the initiative; however, we included sessions with care staff and team managers as part of the process of review and reflection on developing individualised care plans. Initially all parents who were registered at the Family and Child Support Centres were given an open invitation to attend on a specified date. These families included both those who were referred from health or social services, as well as those who self-referred. After the initial sessions we applied a more purposive sampling approach to intentionally capture a diverse range of experiences. For example, we held sessions with mothers from rural and from urban areas, and with fathers/grandfathers, and parents who had attended parenting courses.

### Stage B. The Process of Information Gathering

In total, 41 network-mapping sessions were carried out over 2-years. We included 48 mothers and 12 fathers, all of whom were parents of children with disabilities. The care staff/service delivery personnel at the different centres involved in the PFF programme also completed network-mapping sessions at different points in the cycle of the programme.

The experience of completing the first two rounds of data gathering suggested expanding the sessions to explore the networks of mothers already observed to display different levels of skills in scaffolding their children's behaviours, a key contributor to early childhood development and resilience (11). These skills, including praise, directing attention, responding to needs and interests, were measured using the Observation of Mother-Child Interaction (OMCI), a tool with established reliability and validity in similar social-cultural settings (12). We identified 12 mothers with low level skills levels and 9 mothers with high level skills and invited them to discuss their external support networks.

**Figure 4:**
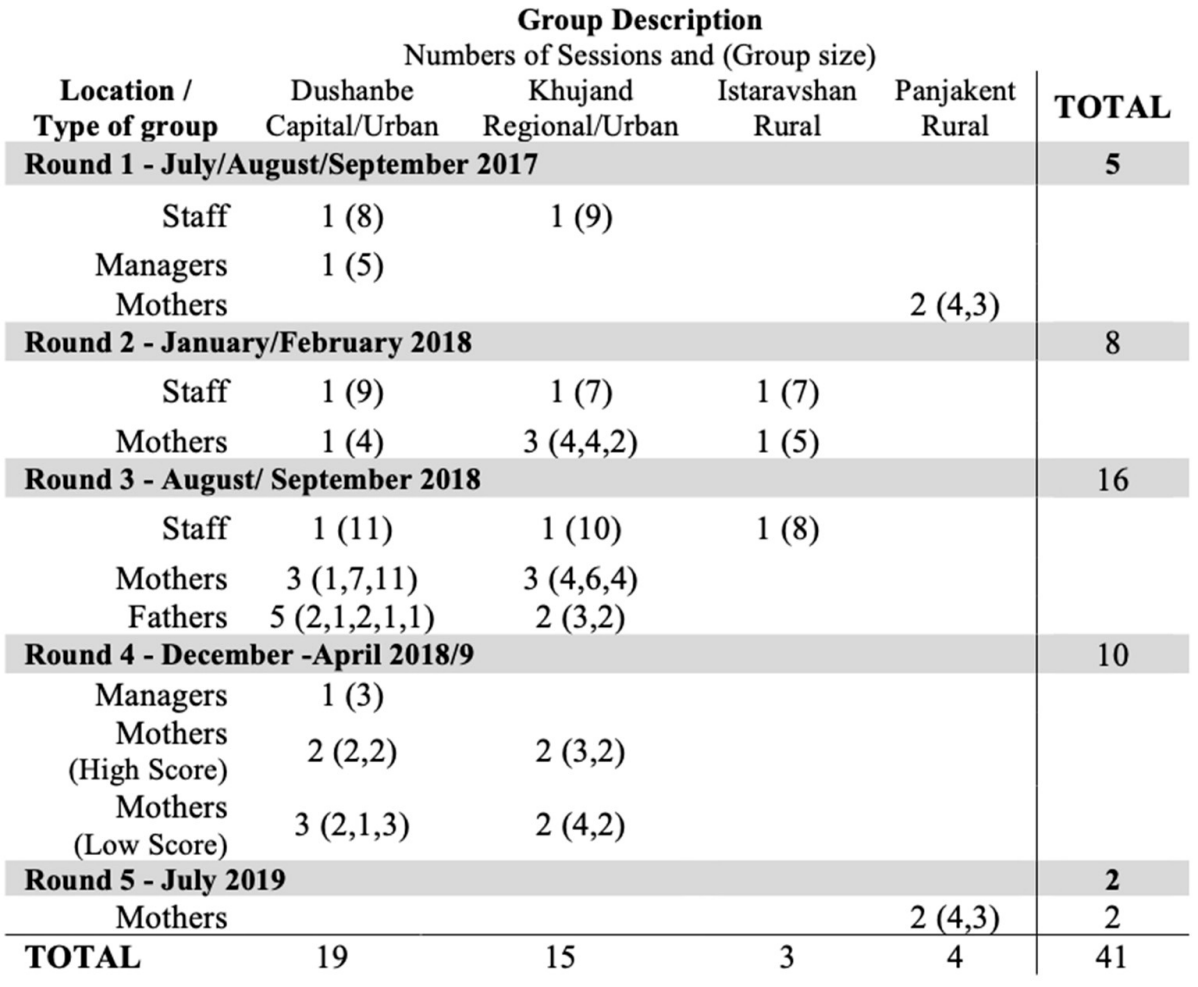
Table of sessions completed.

The sessions are outlined in Figure 4, categorised by number, type and location.

Initially it was exclusively mothers who answered the invitation to attend, with group sizes ranging between 1 and 11. The enthusiasm among mothers to attend these sessions remained consistent. Among mothers who received direct invitations, those with high and low scores on the OMCI, all attended. To include the voice of fathers we also contacted them directly, but struggled to attract more than one or two fathers/grandfathers per session.

Answering the question, “Are participants actively contributing to the sessions?” we established that a group size of between 4 and 6 best generated shared discussions, while smaller groups (1, 2) were useful to explore more specific needs and issues.

### Stage C. The Application of the Knowledge Gained

In this stage the focus of activity was the use of systematic reporting to effectively summarise and share the learning. The implementation team, in reviewing the details of the network-mapping sessions, drew out key takeaways from the maps themselves; the data capture forms (providing quantitative summaries on each characteristic of support); and the accompanying narratives. The team also reviewed the contribution made by each data source in guiding their understanding of the way in which systems of support are experienced.

**Figure 5:**
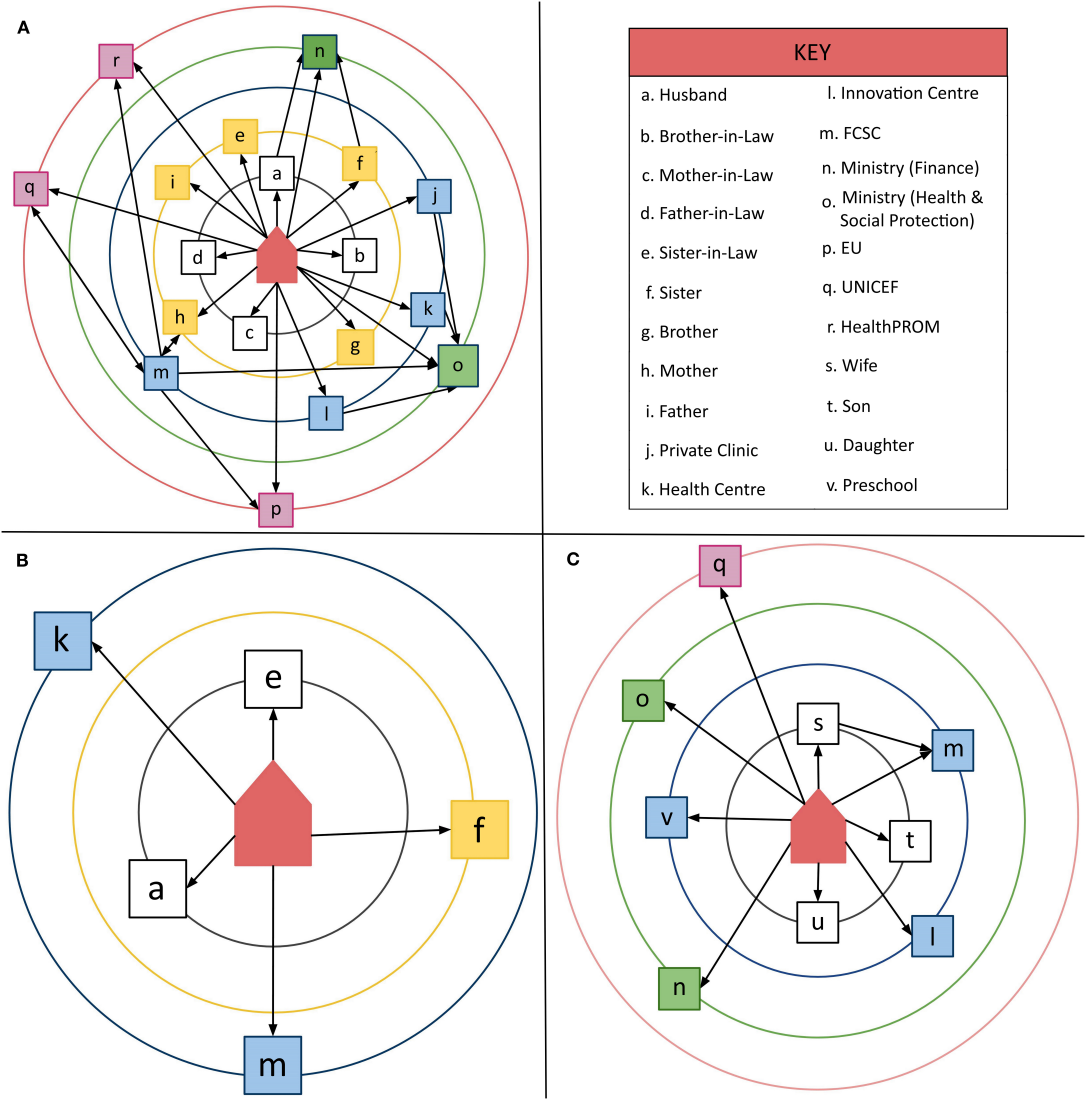
**(A–C)** Graphical representations of 3 diverse network-maps.

The following Figure 5 provides graphical examples of the group network-maps collected. Figure 5A illustrates the relatively complex network that mothers with a high OMCI score. This pattern contrasts with the simpler, less extensive networks of mothers with a low OMCI scores (Figure 5B) and of fathers (Figure 5C). An active community based network exits to support nurturing care, but these network-maps illustrate that it is not accessed equally by all caregivers.

We saw the importance of the immediate family across all study sites. From the mothers' groups we learnt that the immediate family, with its multiple generation households, was consistently the most active source of support, providing direct care to the child, as well as financial and other resources. Mothers highly valued this support, which they ascribed to the social responsibility felt by other family members. One mother said: “*because we are family*” (Mother, Dushanbe). There were exceptions to these positive reports raised by a number of groups. An example was the sister-in-law, who, while resident in the same household, was rated as the least valued member of the support network. Another key figure in the household is the mother-in-law, and assumptions previously held that they were the main influence behind the abandoning of children to institutional care was not supported. Our caregivers rated the importance of the mother-in-law in a more positive way. Exploring the details of different roles within the family, and how they can be encouraged and strengthened needs further exploration.

Even in the early network-maps, mothers identified links to sources of support other than the family, at each of the five levels described in Figure 1. As the activities of the intervention programme rolled out, and in line with programme objectives, there was a shift towards more mothers attending parental skills training, and more mothers drawing on help from more distal sources of support. An important change, when considering children with disabilities, was increased awareness of the support provided for integration into education and the wider community.

The biggest difference across sites was in the strength of the links to more distal providers, in particular the international NGOs. Mothers living in the capital, Dushanbe, were initially far more likely to mention this source of support than those living elsewhere. Following more active engagement in rural areas, network-maps increasingly identified local centres and services as an important provider of support. Few mothers were aware that the centres they attended received support directly from international NGOs. This suggests that the influence of PFF on rural families was more indirect, coming through local service delivery networks.

In early network-maps mothers commonly characterised the father's role as providing resources rather than directly interacting with the child. This observation led us to explore in more detail how fathers understood their role, and how they themselves felt supported as caregivers of a young child in need. We found fathers have far fewer connexions in their support network than mothers. They did not perceive a clear connexion between the services provided and the needs of their children and were generally less aware of which services and activities were available. Some fathers had connected to additional financial resources, but none we interviewed had attended parental training sessions. We did, however, learn from the later network-mapping sessions that, as the PFF programme progressed, mothers saw some positive changes in the contributions made by fathers. Fathers who had attended parental training courses, for example, were reported to have become more supportive and to spend more time with their children. Overall, relationships with the key figures of the husband and mother-in-law were reported by mothers to have improved.

Exploring motivation was a useful way to understand the quality of support caregivers felt they received. Mothers, for example, recognised that the Family and Child Support Centres met a range of social and psychological care needs. They experienced centre staff as socially responsible, listening and responding to individual needs, and motivated to create social change. Before these centres opened, mothers had primarily sought support from medical services, and the onward connexion, through referral to social services, for more social/psychological care was rare. As we explored the differences in how different service providers were valued we came to understand that when mothers feel valued they also accord those service providers greater value. The difference in ratings between different services appears to centre on the presence or absence of shared respect, a central feature of strong relationships.

In all later network-mapping sessions mothers reported having observed significant changes and improvements in their children's speech, behaviour, socialisation skills as well as developing greater independence. They described their children as requiring much less help from others. Mothers attributed this change to having attended the Family and Child Support Centres and valued receiving the services offered free of charge. They also described increased agency, having acquired new knowledge and skills from the centres to better care for their children. As one mother said, “*Many organisations help, they bring us humanitarian aid, but your project is the only one that helps us psychologically*” (*Mother, Panjikent*). The benefit of psychological support was linked to more positive relationships with their children. As another mother said, “*We are now friends with our children. They are more open. They do not fear us and share their feelings and their mistakes*” *(Mother, Khujand). A further mother told us*, “*We became more strong and independent, more confident, more open; we know our rights. Our motto is, ‘Now I can do it'*” *(Mother, Khujand)*.

Exploring the association between social networks and wellbeing was the focus of the comparison of the mothers with high vs. low OMCI scores. Mothers with a high score were able to name people or organisations at all levels. These mothers also recognised specific improvements in their children's progress, reported that their partners showed an increasing understanding of what they could contribute, and were generally aware of the nature of their children's rights. In contrast, mothers with low OMCI scores felt largely unsupported, were less likely to seek help, and were much less aware of the possibility of stimulating changes in their children. They were, nonetheless, grateful for the support provided by the Family and Child Support Centres. The interaction between having a support network to draw upon, and being able to identify and meet the needs of your child, will be an important focus for exploration in case management within the programme in the future.

Overall, while the range of network sources mentioned by participants did not expand over the 2-year project period, we did observe shifts towards greater access to sources of support outside of the family. Another important change was the more positive relationships reported with those providing support, both inside and outside the immediate family. As one mother said in later network-mapping session, “*We learned how to build relationships with others*” (*Mother Khujand*).

When asked to share their feelings about participating in the network-mapping sessions, parents expressed their gratitude for being listened to, and having their views acted upon. They also valued the opportunity to learn from and develop friendships with each other. This shared learning enabled them to expand further their support networks as they became aware of the availability of other rehabilitation centres, of the existence of inclusive schools or kindergartens, as well as of the financial support they were entitled to from the state. The network-mapping sessions became a part of the network of support that brings people together to share information, experiences and knowledge.

Further evidence of the value of the measurement process in decision-making was observed through the sessions held with staff and management groups. Sharing the information collected from parents' groups provided the opportunity to reflect on service delivery strengths and weaknesses and to identify potential targets for improvement. In these sessions the teams also reviewed the resources available in the nurturing care system, from the perspective of the service delivery system. They also examined gaps in their current links to these resources to suggest where communication channels could be improved. While the provision of training in parental skills featured more regularly in later network-maps of the mothers, limitations remained in the inclusion in this and other services of fathers. Another concern was that few families were aware of the child protection services offered by local government, highlighting the need to strengthen the capacity building initiatives provided by the PFF programme for other services. Stronger ties with local government systems, for example, were identified as a priority.

## Discussion

### Stage D. The Review of Policy and Practise

The focus of this stage was to reflect on the network-mapping process and generate recommendations that guide the continuous strengthening of a community based approach to nurturing care. In our application of network-maps we found a functioning tool that contributes to decision-making in multiple aspects of implementation (9). Network-mapping enabled a detailed examination of the complexity of support, expanding beyond the ‘who' and the ‘what' to better understand the influence of underlying values and perceived motivation. The nature and degree of social interactions were captured, identifying the strengths in the system and gaps where further strengthening could create more active parent and child inclusion.

Using this tool has brought to life Bronfenbrenner's EST by superimposing representations of real social interactions and influences upon a generic nested model. The ecology of support systems in Tajikistan, described through the network-maps, initially pointed to the relevance of the nested structure described by Bronfenbrenner (8). Through an exploration of values and motivation we observed that the extended family connected to the primary caretaker blurred the boundary between the micro- and meso- levels. Although more distal connexions were initially less visible and less valued, there was a shift in this conceptualisation as the Family Support Centres rolled out their services. Not only were the skills and confidence of the immediate caregivers, both fathers and mothers, strengthened, but the external support system also became more valued. This suggests that a networked rather than nested approach to understanding and describing the ecology of support is more descriptive in this context. A network approach also better reflects the more equally distributed decision-making that resulted from the empowerment activities and is even more relevant as a community-based support system becomes more embedded.

This adds an extra dimension of “networks” to Bronfenbrenner's “nested” model. As Neal and Neal (13) also suggest “*by focusing attention on patterns of social interaction, the networked model offers the possibility of using the precise tools of social network analysis to move EST from a theory to a method*.” The network-mapping tool we adapted for use in Tajikistan is an example of such a tool, making the connexion from theory to method. A network approach avoids a hierarchical framework to programme design, providing an interactional dynamic framework for connecting the theory-practise-policy cycle.

One of the primary means through which this tool has connected theory to method has been to provide families with a voice, and involve them directly in monitoring services. We have learnt of the impact of the project on their daily lives. We have heard both positive and negative feedback in their own words. This dialogue has strengthened the programme, guided the improvement of services and changed the course of the project. It has enabled us to learn more about the needs of specific individuals and discrete sub-groups of the more vulnerable. This shift in thinking, and in the details of the process of implementation, has influenced review of our theory of change. Accordingly, project managers focused more on a rights-based approach that recognises and counters the sources of social exclusion.

For example, among mothers whom we observed as providing their children with limited stimulation and responsiveness, those scoring low on the OMCI, we also found very limited networks of support. The network-mapping sessions helped us learn about the social needs of these mothers, and deepened the thinking around how the programme can link social with psychological support. In fact the experience of taking part in network-mapping prompted some mothers struggling to care for their children to opt into project activities and join parent to parent support groups from which they had previously felt excluded.

Discussions with fathers exposed the sparseness of their networks and their lack of involvement in parenting activities. Project managers came to understand better the challenges fathers face in integrating effectively into the childcare system, prompting the design of activities specifically for these key caregivers. We also observed that strengthening fathers' skills and parenting knowledge, leading to greater participation in childcare, served to reduce gender inequality as well as contributing to Sustainable Development Goal 5.

Through this process we have learnt of the association between being valued and actively engaging with services. This narrative was expanded upon by centre staff, who felt that health services lacked an awareness of both the complex needs of children with disabilities and of the network of services available to meet them. We were also concerned by the limited role of State child protection agencies in the network-maps drawn. The network-mapping process highlighted these gaps, and suggested the need for further outreach to professional groups.

For both parents and professionals, we found that network-mapping helps people orient themselves in *place*- by understanding where they fit into the big picture; and in *time*- by helping people see their purpose and direction for future development. We were struck by the insight network-maps gave participants about their own motivation, as well as understanding their place in a wider network. We repeatedly heard how participants felt included, and for team members, this indicated that network-mapping is a tool for team cohesion. Network-mapping sessions made an important contribution to the understanding of the bigger picture of the network of support for all involved the process. In addition to knowledge building, this tool also shaped the skills required to improve community outreach through the inclusion of caregivers as equal partners.

Network-mapping sessions therefore contributed to a beneficiary-led review of how services should change to better meet individual and group needs. The detail of information collected improved the awareness of need, and informed the development of individual care plans, training programmes and the structure of service delivery. Other applications in diverse settings of similar methods have similarly found both the concept and the process of mapping networks valuable for creating individualised and interactive care plans (14, 15).

The use of a network-mapping process has generated detailed information on the development of a systematic and rigorous monitoring, evaluation and learning system. The feedback from the team has highlighted the value of more qualitative information and visual displays in earlier stages of development. The maps and narratives were also influential when used in dialogue with relevant ministries. More extensive experience is required before quantitative data is usefully interpreted. Sensitivity is needed in selecting appropriate methods of data capture and sharing in order to help effectively involve individuals, communities, organisation and institutions to meet their needs and create lasting change (16–18).

## Recommendations

In reflecting on the learning derived from the network mapping process the following recommendations were generated by the senior management team.

- **Informed, responsive design**. This methodology was valued as a key activity in monitoring, informing, and communicating family needs, and system strengths and weaknesses. As the programme progresses the team will continue to share the network-maps and the details of the conversations they generate to cement communication systems between the different parts of the network. This sharing will generate ideas on how to improve the system and services still further, while also uniting parents through peer support groups. In addition, network-mapping generated detailed information useful for advocacy and policy development. As the programme is extended to other regions, network-mapping will be an essential monitoring and evaluation tool to help us effectively reach individuals and communities, and meet their needs. As we apply this tool in future settings we will focus on integrating this work more directly into the structure of delivery.
- **Individualised Services**. Embedding the procedural connexion between network-mapping and case management in future delivery systems will create a more international connexion between lessons learned around a family's social interactions and the care plans made. This will require building capacity within the service delivery team to carry out individual network-mapping sessions.
- **Flexible Application**. Expand the use of the methodology to explore more specific foci in the networks of support for childcare.
  - An important network to expand knowledge of is the child's learning environment, both formal and informal, mapping opportunities for play, language stimuli and inclusion.
  - Another key area of focus will be the social connexions that support the adaptation of child protection policies and practise to a community based childcare framework.
  - We need to explore the needs of specific subgroups in more detail, in particular, mothers unsupported by immediate family.
  - Address the issue of support networks from different perspectives, from the micro, meso, and macro levels.
- **Utilise an integrated MEL process**. The network-mapping process illustrates a practical application of the aspirations of Measurement for Change, that advocate for a continuous process of consultation, measurement, review and reflection to build capacity to achieve a sustainable and scalable implementation process (19). As we apply this tool in future settings we will focus on integrating this work more directly into the structure of delivery. To do so effectively and with quality we recommend that those new to the process will need to refine their facilitation skills and familiarity with the material over time. They should carry out at least five practise sessions before starting to gather information in a more rigorous, systematic way. They will also need guidance in reading, analysing and interpreting data.

## Acknowledgements of Limitations, Conceptual, and Methodological Constraints

The team involved in this programme, largely applied practitioners, began with limited experience of managing and utilising integrated monitoring and evaluation systems. As the project progressed the value of the information gathered from the network-maps grew alongside an increasing appreciation of rigorous and systematic data systems. The implementation team built an awareness of the interconnection between developing the skills to track and measure change and the value of the information collected to improve communication and decision-making. The process of transition from data being used to deliver instructions to a collaborative learning process was achieved. Observable progress towards creating a link between a systematic process to identify and track participants and improved service delivery was also achieved. A longer time frame is required to build trust and demonstrate the value of generating hard data on programme impact.

The families included in this process were drawn from among those caring for children with disabilities. The networks observed may not reflect those of other populations of families in need.

## Data Availability Statement

The raw data supporting the conclusions of this article will be made available by the authors, without undue reservation.

## Ethics Statement

The studies involving human participants were reviewed and approved by Tajikistan Ministry of Health and Social Protection of the Population. The participants provided their written informed consent to participate in this study. Written informed consent was obtained from the individual(s) for the publication of any potentially identifiable images or data included in this article.

## Author Contributions

All authors contributed equally to the development of the process presented in this paper.

## Conflict of Interest

The authors declare that the research was conducted in the absence of any commercial or financial relationships that could be construed as a potential conflict of interest.

## Footnotes

^1^www.healthprom.org

^2^Index Mundi (2019) Tajikistan Urbanization. Available online at: https://www.indexmundi.com/tajikistan/urbanization.html (accessed May 9, 2020).

^3^https://www.ohchr.org/EN/NewsEvents/Pages/DisplayNews.aspx?NewsID=24451&LangID=E

^4^*https://netmap.files.wordpress.com/2008/06/net-map-manual-long1.pdf*

^5^For example forms please contact corresponding author.

^6^Tajikistan's average elevation above sea level is 10,455 feet, being the third highest country after Bhutan and Nepal. https://www.worldatlas.com/articles/countries-with-the-highest-average-elevations.html
